# Profiling of Cxcl12 Receptors, Cxcr4 and Cxcr7 in Murine Testis Development and a Spermatogenic Depletion Model Indicates a Role for Cxcr7 in Controlling Cxcl12 Activity

**DOI:** 10.1371/journal.pone.0112598

**Published:** 2014-12-02

**Authors:** Birgit Westernströer, Nicole Terwort, Jens Ehmcke, Joachim Wistuba, Stefan Schlatt, Nina Neuhaus

**Affiliations:** 1 Centre of Reproductive Medicine and Andrology, Institute of Reproductive and Regenerative Biology, University Muenster, Muenster, Germany; 2 Central Animal Facility of the Medical Faculty, University Muenster, Muenster, Germany; National Cancer Institute, United States of America

## Abstract

In mice the chemokine Cxcl12 and its receptor Cxcr4 participate in maintenance of the spermatogonial population during postnatal development. More complexity arises since Cxcl12 also binds to the non-classical/atypical chemokine receptor Cxcr7. We explored the expression pattern of Cxcl12, Cxcr4 and Cxcr7 during postnatal development in mouse testes and investigated the response of Cxcl12, Cxcr4, Cxcr7 and SSC-niche associated factors to busulfan-induced germ cell depletion and subsequent recovery by RNA expression analysis and localization of the proteins. In neonatal testes transcript levels of *Cxcl12*, *Cxcr4* and *Cxcr7* were relatively low and protein expression of Cxcr7 was restricted to gonocytes and spermatogonia. During development, RNA expression of *Cxcl12* remained stable but that of *Cxcr4* and *Cxcr7* increased. Cxcr7 was expressed in germ cells located at the basement membrane of the seminiferous tubules. In adult testes, transcript levels of *Cxcl12* were highest while the localization of Cxcr7 did not change. Following germ cell depletion, a significantly increased expression of *Cxcl12* and a decreased expression of *Cxcr7* were observed. Germ cells repopulating the seminiferous tubules were immunopositive for Cxcr7. We conclude that Cxcr7 expression to be restricted to premeiotic germ cells throughout postnatal testicular development and during testicular recovery. Hence, the spermatogonial population may not only be simply controlled by interaction of Cxcl12 with Cxcr4 but may also involve Cxcr7 as an important player.

## Introduction

In mammalian testes, the unipotent spermatogonial stem cells (SSCs) reside within specialized microenvironments called ‘niches’, essential for the regulation of stem cell self-renewal and differentiation. The Sertoli cells, forming the blood-testis barrier (BTB), and the basal lamina are the structural components of this SSC niche [1]–[3]. Sertoli, peritubular as well as Leydig cells provide extrinsic factors that are essential for migration, stem cell retention, and for the regulation of SSC functions [4]–[9]. Amongst such factors are the colony stimulating factor-1 (Csf-1) produced by Leydig cells as well as the glial cell-line-derived neurotrophic factor (Gdnf), which is secreted by Sertoli cells from birth through adulthood. Gdnf regulates the proliferation and survival of undifferentiated spermatogonia [9]–[12].

Interestingly, recent studies have revealed that the chemokine (C-X-C motif) ligand 12 (Cxcl12) is involved in the postnatal maintenance of the SSC pool in mouse testes [13]–[15]. While the chemokine is a product of the Sertoli cells only, the C–X-C chemokine receptor type 4 (Cxcr4) is expressed by spermatogonia, Sertoli and interstitial cells [14]–[16]. The functional role of the Cxcl12/Cxcr4 interaction within the adult mouse testis has been investigated *in vitro* and it was suggested that the ligand/receptor pair is involved in SSC propagation but prevents SSC differentiation [15]. In addition, it has been experimentally shown by germ cell transplantation experiments, that in congenitally infertile W/W^v^ mouse testes an increased expression of Cxcl12 by Sertoli cells leads to an elevated/enhanced colonization of seminiferous tubules by Cxcr4 positive SSCs. Based on these studies it was concluded that the Cxcr4 mediated action of Cxcl12 also plays a role for the homing and colonization process of SSCs into their niches [14], [15]. The stimulation of colonization and migration of stem cells into their niches via Cxcr4 is also known to play a role during hematopoiesis [17], [18]. Furthermore, the interaction between Cxcl12 and Cxcr4 is also required for the migration as well as for the maintenance of primordial germ cells (PGCs) in mice [19], [20].

Moreover, the C–X-C chemokine receptor type 7 (Cxcr7) was identified in zebrafish as an alternative receptor for Cxcl12 which created more complexity to this rather simple model regarding the regulation of PGC migration [21]–[24]. In contrast to Cxcr4, Cxcr7 belongs to the group of atypical chemokine receptors [25]–[28] and the interaction with Cxcl12 rather results in an internalization of the chemokine without inducing downstream signaling. Consequently, the action of Cxcr7 rather results in the generation of a Cxcl12 gradient thereby facilitating the directed migration of PGCs in zebrafish [21], [22], [29]. So far, transcripts of *Cxcr7* were detected in testes from adult rats and humans [30], [31]. More specifically, Eva *et al.* 1993 reported previously a cloning and sequencing analysis of *Cxcr7* (also called *Rdc1*) in a rat forebrain library and demonstrated mRNA expression of *Cxcr7* in lung, kidney and testes of adult rats by northern blot analysis [30]. Twenty years later, McIver *et al.* 2013 investigated normal and pathological human testes and found that *CXCR7* transcripts in seminomas and non-seminomas to be significantly lower compared to the matched control tissue [31]. However, neither the localization nor the function of this receptor during fetal and postnatal mammalian germ cell development has been investigated. Moreover, to date the capacity of the Cxcl12/Cxcr4/Cxcr7 axis to support the re-establishment of spermatogenesis following an induced germ cell loss, remains largely unknown.

In the present study, we investigated the expression pattern of Cxcr7 during postnatal germ cell development in mouse testes. Moreover, we evaluated the response of several niche-associated factors, including the chemokine Cxcl12 as well its receptors Cxcr4 and Cxcr7 to experimental spermatogonial depletion and subsequent repopulation.

## Materials and Methods

### Ethics statement

NMRI mice at the age of 1, 7, 14, 21 days *post-partum* (d*pp)* and adult animals (>37 d*pp*; between 6 and 21 weeks of age) were purchased from the Central Animal Facility of the Medical Faculty (Muenster, Germany). Mice were maintained under a twelve hours light/dark regime, with pelleted food and water available *ad libitum*. All experimental protocols in this paper were approved by the regional/state authority “Landesamt für Natur, Umwelt und Verbraucherschutz Nordrhein-Westfalen” (State Agency for Nature, Environment and Consumer Protection North Rhine-Westphalia) (animal license No. 87.5104.2010.A244, LANUV NRW, Germany).

All procedures on mice were performed in accordance with the granted procedures and 3R guidelines optimizing animal welfare. The busulfan treatment of mice was performed as outlined below in the animal protocol to minimize suffering. Mice were sacrificed by cervical dislocation under KX anaesthesia (ketamine/xylazine solution in saline; i.p.; Sigma Aldrich, K-113, Steinheim, Germany).

### Tissue collection for characterization of Cxcl12 and its receptors Cxcr4/Cxcr7 during testicular development

**Figure 1:**
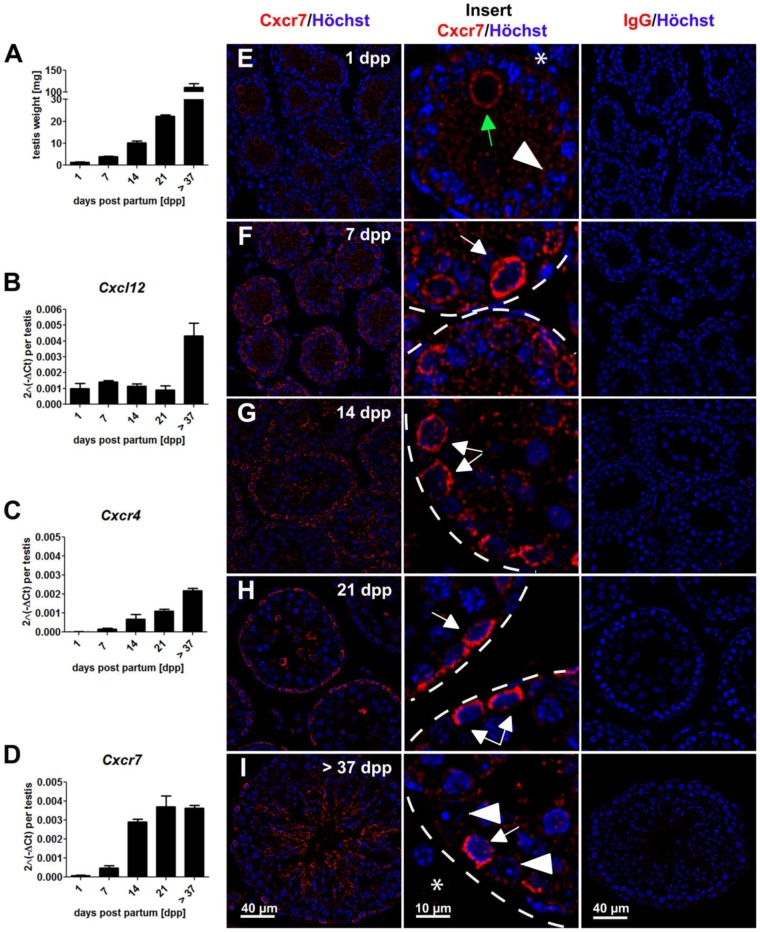
Expression pattern of Cxcr7 during testicular germ cell development. Changes in testicular weight (A) as well as changes in transcript levels of *Cxcl12* (B), *Cxcr4* (C) and *Cxcr7* (D) during postnatal development in mouse testes ((1d*pp* and <37d*pp* (n = 3); 7d*pp*, 14 d*pp*, 21 d*pp* (n = 4)), 2 ^(−ΔCt)^ per testis, relative to *luciferase*). Results are shown as mean ± SD. Representative images showing immunofluorescence stainings for Cxcr7 (red) and Hoechst (blue) on days 1, 7, 14, 21,>37 of postnatal testicular development (first column, E–I). Within the inserts (second column) expression of Cxcr7 was observed in gonocytes (green arrow), spermatogonia (white arrows) and interstitial cells (asterisks). Sertoli cells are indicated with white arrowheads. Results of the respective negative controls using nonspecific IgG antibodies are shown in the right column (IgG/Hoechst). Scale bars represent 10 µm and 40 µm, respectively.

Mice aged 1 (n = 3), 7, 14, 21 d*pp* (n = 4 per age group) and adult animals (n = 3; between 19 and 21 weeks of age) were sacrificed. One testis from each animal was weighted (testis weights increased gradually from 1.3±0.5 mg on day 1 to 111.8±15.2 mg on day>37 (Fig. 1A)) and snap-frozen for RNA expression analysis, whereas the other testis was fixed for 6 hrs at room temperature (RT) in 4% paraformaldehyde (PFA) for immunohistochemical analysis.

### Gonadotoxic treatment

Butane-1,4-diyl dimethanesulfonate (busulfan) was used as a cytotoxic agent to induce germ cell loss in the testes of adult mice [32], [33]. Busulfan powder (Sigma Aldrich, B2635, Steinheim, Germany) was dissolved in dimethylsulfoxid (DMSO, Sigma Aldrich, D5879, Steinheim, Germany). Adult male NMRI mice (6 weeks old) were either given a single injection (i.p.) of DMSO (vehicle control group, n = 50) or of busulfan at a concentration of 38 mg/kg (treated group, n = 50). On days 1, 3, 7, 21 and 28 post treatments, ten animals per time point from both groups were killed as described above and evaluated. In addition, to control for potential DMSO effects, eight mice received a single injection (i.p.) of 0.9% saline (sham control group) and were killed 24 hrs later. Sham-treated NMRI mice had a mean testis weight of 96±4.5 mg. DMSO treatment alone had no apparent effect on the testicular weight, however, a significant reduction was detected on days 21 (57.0±13.6 mg) and 28 (41.6±3.6 mg) after busulfan treatment (Fig. S1).

### Tissue collection following gonadotoxic treatment

Following treatment, mice were sacrificed and one testis from each animal was weighed and snap-frozen for RNA expression analysis, whereas the other testis was divided into two parts, one of which was fixed in Bouin's solution and the other in 4% PFA for periodic acid Schiff's (PAS) staining and immunohistochemical analysis. While busulfan treatment was effective in the majority of animals, two mice showed no decrease in testicular weight and histological analysis revealed normal spermatogenesis. Therefore, these animals were excluded from the study.

### RNA isolation and relative gene expression analysis

Per testis sample, 20 ng luciferase mRNA (Promega, L4561, Mannheim, Germany) were added [32], before RNA extraction was performed using Ultraspec (Biotecx, Houston, USA) and genomic DNA was removed by DNase treatment (DNA-free Kit, Ambion, Darmstadt, Germany). Using 1 µg of total RNA, cDNA was generated employing SuperScript II Reverse Transcriptase (Invitrogen, Darmstadt, Germany) and random hexamer primers (Promega, Mannheim, Germany).

Quantitative real-time PCR (qRT-PCR) analysis was performed using SYBR Green technology. For relative quantification of SSC marker genes and niche-associated factors, the following genes were selected based on the current literature (Table S1) *Lin28a* (undifferentiated spermatogonia), *Ddx4* (general germ cell marker), *Cxcl12*, *Gdnf*, *Amh* (Sertoli cells), *Erm, Itgb1, Cxcr4* (Sertoli cells and spermatogonia) and *Cxcr7* (to be determined). Specific primers were designed using Primer Express 3.0 Software. Optimal primer concentrations, primer specificity and PCR efficiency were evaluated following the Power SYBR Green PCR User Guide (Applied Biosystems, Darmstadt, Germany). Primer sequences, localization of proteins within the mouse testis and the respective references are summarized in Table S1. For qRT-PCR analyses, cDNA was diluted 1∶10 and 2 µl were used for each 20 µl PCR reaction with Power SYBR Green Mastermix (Applied Biosystems, Darmstadt, Germany). The PCR programme consisted of initial steps of activation and denaturation which were run once for 10 min at 95°C, followed by 40 cycles of denaturation (15 sec at 95°C), annealing and elongation (1 min at 60°C). qRT-PCRs were run on the StepOnePlus (Applied Biosystems, Darmstadt, Germany) and were subsequently analyzed using the StepOne software 2.2 (Applied Biosystems, Darmstadt, Germany).

To calculate relative expression levels of *Cxcr7*, *Cxcr4* and *Cxcl12* during postnatal germ cell development in mice, the 2 ^(-ΔCT)^ method was applied [34]. Rather than using an internal reference gene, luciferase was employed as an external standard, facilitating the calculation of expression levels per testis, as previously described [32]. Therefore, this approach allowed for a direct comparison of expression levels between tissues of different weights and cellular composition.

Following gonadotoxic treatments, the 2 ^(-ΔΔCT)^ method was applied [34] using luciferase as external standard and sham control animals (n = 8) as calibrator. As Leydig and Sertoli cell numbers are not affected by busulfan and DMSO treatment, no further corrections regarding the transcript levels per testis were required [32].

### Histological analyses and repopulation index

Testes fixed in Bouin's solution were transferred into 70% ethanol the next day, whereas PFA fixed tissues were placed into 30%, 50% and 70% ethanol (30 min each), respectively. Routine paraffin-embedding was performed and testes were sectioned at 3 µm using a Leica SM2000R microtome (Leica, Wetzlar, Germany). For histological evaluation, sections were stained using PAS staining followed by haematoxylin counterstaining [35].

Following gonadotoxic treatment and germ cell depletion, the repopulation index (RI) was determined on day 28 after busulfan treatment (RI_28d_, n = 4). In each testicular section, 25 seminiferous tubules were evaluated for the presence of germ cells and based on these results the percentage of seminiferous tubules showing germ cells that had reached the spermatogonial stage or later was determined [36].

### Immunohistochemical stainings on testicular tissue sections

Paraffin was removed from sections using Pro Taqs Clear (Pro Taqs Clear, Cat. No. 4003011, Quartett Immunodiagnostika & Biotechnologie, Berlin, Germany) and sections were subsequently rehydrated in a decreasing ethanol series. After rinsing with distilled water and Tris-buffered saline (TBS), sections were placed into citrate buffer (pH 6) and were heated in the microwave for 12 min. After cooling to RT, sections were washed in TBS and non-specific peroxidases were blocked with 3% (v/v) H_2_O_2_ for 15 min at RT. In order to block non-specific binding sites, sections were incubated with 25% chicken serum in TBS containing 0.5% (w/v) bovine serum albumin (BSA) for 30 min at RT. Subsequently, primary antibodies against LIN28a (rabbit polyclonal anti-LIN28a, A177 Cell Signaling, dilution of 1∶50, Darmstadt, Germany) and DDX4 (rabbit polyclonal anti-DDX4, ab-13840 Abcam, dilution of 1∶200, Wiesbaden, Germany) were applied and sections were incubated in a humid chamber at 4°C overnight. Incubation with the corresponding immunoglobulin G (IgG) fractions served as negative controls. After three washes in TBS, primary antibodies were detected using secondary antibodies labeled with biotin and tertiary antibodies with a streptavidin-conjugated horseradish peroxidase (S5512, Sigma-Aldrich, Steinheim, Germany). Finally, staining was visualized using 3,3′-diaminobenzidine as chromogen (D4168, Sigma-Aldrich, Steinheim, Germany), and hematoxylin as counterstain.

### Immunofluorescence stainings on testicular tissue sections

Sections were dewaxed and rehydrated as described above. After rinsing with distilled water and phosphate-buffered saline (PBS), sections were incubated with 25% goat serum in TBS containing 5% (w/v) bovine serum albumin (BSA) for 30 min at RT. Subsequently, primary antibodies against CXCR7 (rabbit polyclonal anti-CXCR7, Abcam, dilution of 1∶400, Cambridge, UK), CXCR4 (rat monoclonal anti-CXCR4, R&D System, dilution of 1∶20, Wiesbaden, Germany), LIN28a (rabbit polyclonal anti-LIN28a, A177 Cell Signaling, dilution of 1∶50, Darmstadt, Germany) and SALL4 (mouse monoclonal anti-SALL4, Abcam, dilution of 1∶150, Cambridge, UK) were applied and sections were incubated in a humid chamber at 4°C for 60 min. The specificity of the anti-CXCR7 antibody was confirmed immunohistochemically using peptide competition (Fig. S2A–D). For this, the human GPCR RDC1 peptide was applied in a ratio of 1∶1 with the anti-CXCR7 antibody to testicular tissue sections (14 d*pp*, incubation 1 hr at RT, Abcam, Cambridge, UK). Incubation with corresponding immunoglobulin G (IgG) fractions was used as negative control. Following two PBS washing steps the appropriate Alexa fluor 488-linked or 546-linked secondary antibodies, diluted in TBS/5% BSA, were applied for 45 min at RT in the dark. Cells were counter-stained with Hoechst for visualization of nuclei (33258, Sigma-Aldrich, Steinheim, Germany).

### Statistical analysis

Statistical analyses were performed using GraphPad Prism 5 (GraphPad Software, USA). For the gonadotoxic treatment study, significant differences were determined using the Mann-Whitney test and significance levels are indicated as *, P = 0.01 to 0.05; **, P = 0.001 to 0.01 or ***, P<0.001.

## Results

### Cxcl12, Cxcr7 and Cxcr4 expression during testicular germ cell development in mice

Transcript levels of the chemokine *Cxcl12* per testis remained constant from birth until day 21 and increased only in adult animals (4-fold, Fig. 1B). mRNA levels of the chemokine receptors *Cxcr4* (Fig. 1C) and *Cxcr7* (Fig. 1D) were low at birth and increased on day 21 *pp* and in adult animals (>37 days d*pp*), respectively.

Immunofluorescent staining revealed that gonocytes were positive for Cxcr7 on day 1 *pp* (Fig. 1E, Insert). On days 7 (Fig. 1F), 14 *pp* (Fig. 1G) and 21 *pp* (Fig. 1H), germ cells located at the basement membrane were positive for the receptor Cxcr7. Additionally, on days 14 and 21 *pp* differentiated germ cells were also Cxcr7-positive. Finally, in testes at 37 d*pp* (Fig. 1I), some interstitial cells and germ cells at the basement membrane as well as spermatids stained positive for Cxcr7. The cellular distribution pattern of Cxcr7 during testicular germ cell development in mouse testes is summarized in Table S2. Double immunohistochemical stainings showed that Cxcr7 and Sall4 co-localize and are expressed in spermatogonia on days 7 (Fig. S2E–G), 14 (Fig. S2I–K) and>37 pp (Fig. S2M–O).

**Figure 2:**
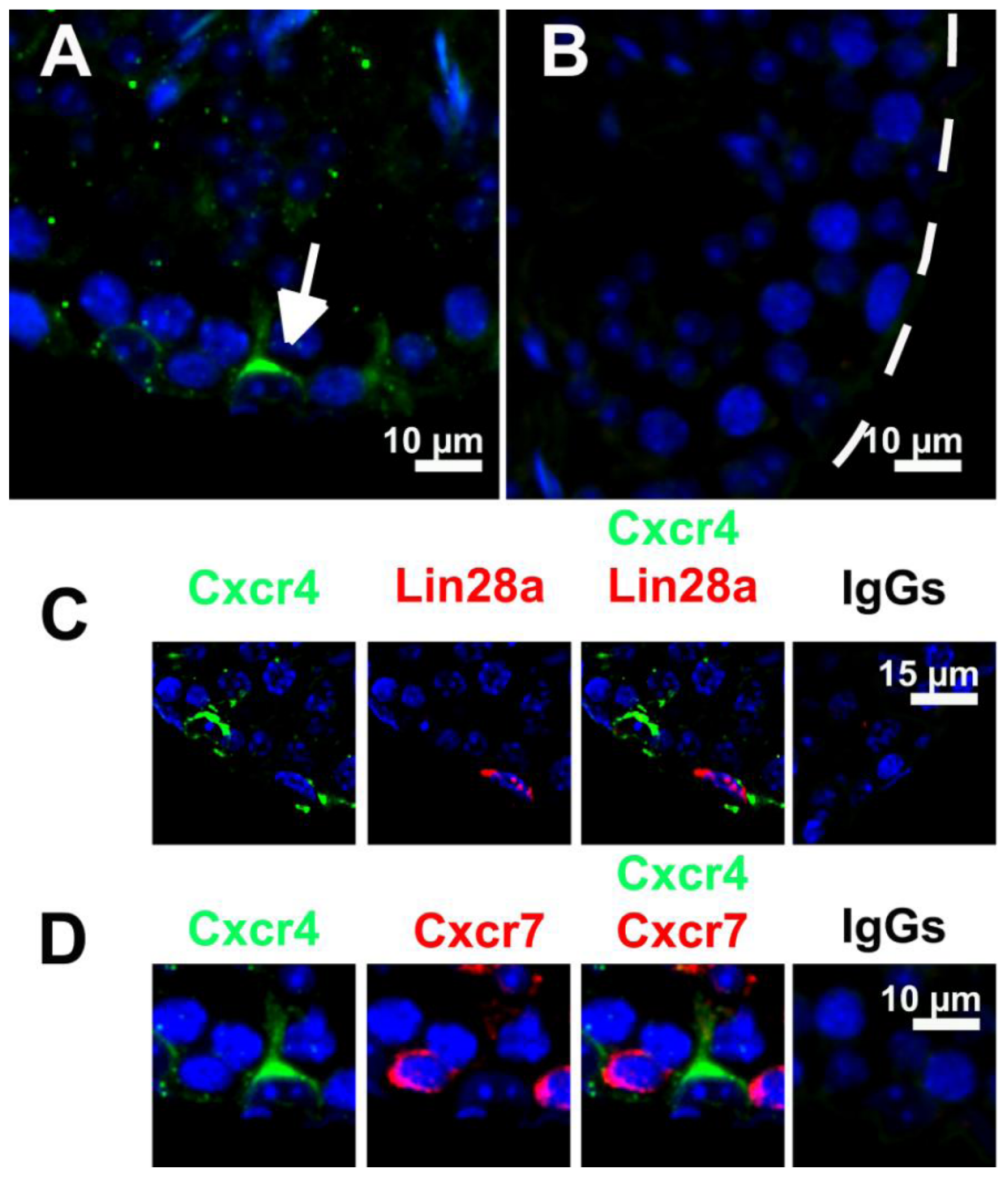
Cxcr4 and Cxcr7 expression in adult mouse testes. Representative images show immunofluorescence staining for Cxcr4 (green, A) in adult mouse testes. Cxcr4 is expressed by testicular cells at the basement membrane (arrow) of seminiferous tubules (indicated by dotted lines). Co-stainings revealed no Cxcr4 expression in Lin28a-positive (C) and Cxcr7-positive (D) spermatogonia. Incubation with corresponding IgG antibodies was used as negative control (B, C and D right column). All sections were counterstained with Hoechst (blue). Scale bars represent 10 and 15 µm, respectively.

In testes of adult mice, the chemokine receptor Cxcr4 was detected in testicular cells located at the basement membrane (Fig. 2). Some interstitial cells were also Cxcr4-positive (data not shown). Neither Lin28a-positive (Fig. 2C) nor Cxcr7-positive (Fig. 2D) spermatogonia co-expressed Cxcr4.

### Histological analysis after busulfan treatment

**Figure 3:**
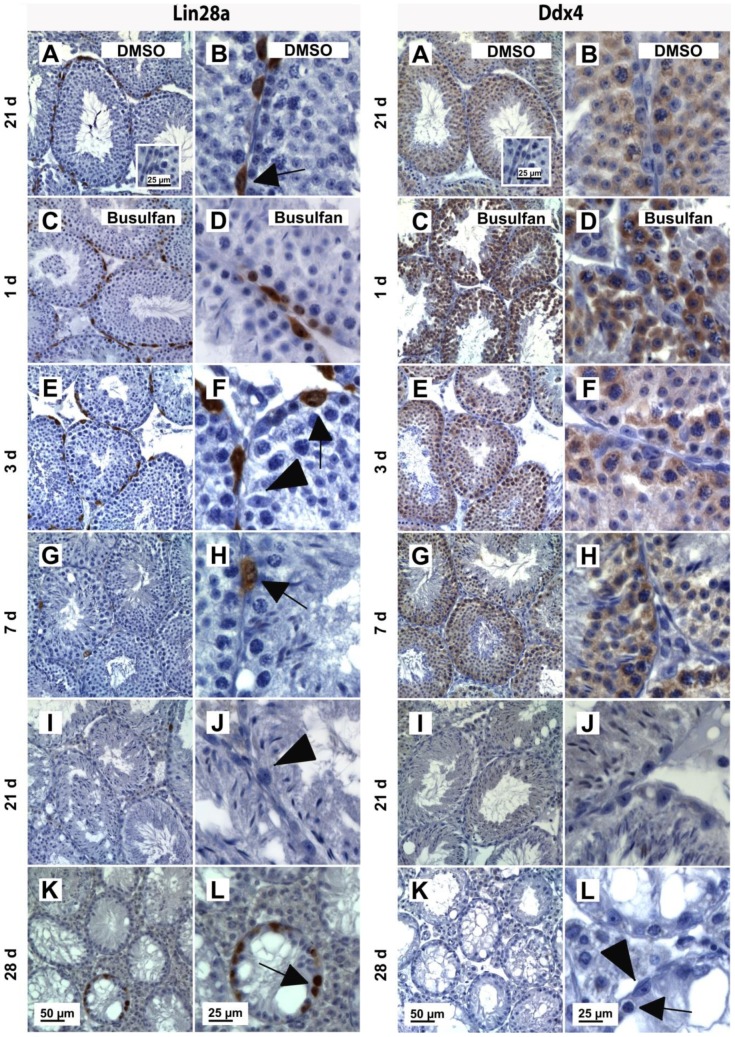
Expression of Lin28a and Ddx4 in adult mouse testes following busulfan treatment. Representative images showing immunohistochemical stainings for Lin28a (left column) and Ddx4 (right column) on testis sections of DMSO (A, B) and busulfan treated (C–L) adult mice. For Lin28a and Ddx4 stainings on days 1 (C), 3 (E), 7 (G), 21 (I) and 28 (K) after busulfan treatment higher magnifications are shown for each time point (D, F, H, J, L). Germ cells are indicated by arrows and Sertoli cells by arrow heads. As negative control, stainings with nonspecific IgGs were performed (A; Insert). Scale bars represent 25 µm and 50 µm.

Histological analysis of PAS stained testicular tissue (Fig. S3) revealed that the injection of DMSO alone had no apparent effect on the somatic or the germ cell population (Fig. S3A–E). In contrast, busulfan treatment resulted in a progressive germ cell loss (Fig. S3F–O). Immunohistochemical stainings furthermore showed that Lin28a positive undifferentiated spermatogonia were located at the basal membrane of the seminiferous tubules until day 3 after treatment (Fig. 3; left column; C, E; Inserts: D, F). On days 7 and 21 however, the majority of tubules were depleted of Lin28a positive cells (Fig. 3; left column; G and I; Inserts: H and J). Finally, on day 28 after treatment, Lin28a expression was observed in several seminiferous tubules showing repopulation of spermatogonia (Fig. 3; left column; K; Insert: L). Immunohistochemical analyses for Ddx4 showed positive spermatocytes and round spermatids in the testes of busulfan treated mice on days 1, 3 and 7 (Fig. 3; right column; C, E, G; Inserts: D, F and H). 21 days after busulfan treatment, Ddx4 expression was still present in some seminiferous tubules containing residual round spermatids (Fig. 3; right column; I; Insert: J) and after 28 days, very few Ddx4 positive cells were detected (Fig. 3; right column; K; Insert: L). Subsequent quantification of those seminiferous tubules containing spermatogonia 28 days after busulfan treatment revealed a repopulation index of RI_28d_ = 0.2±0.2%.

### Relative expression of germ cell- and SSC niche-associated factors following cytotoxic treatment

**Figure 4:**
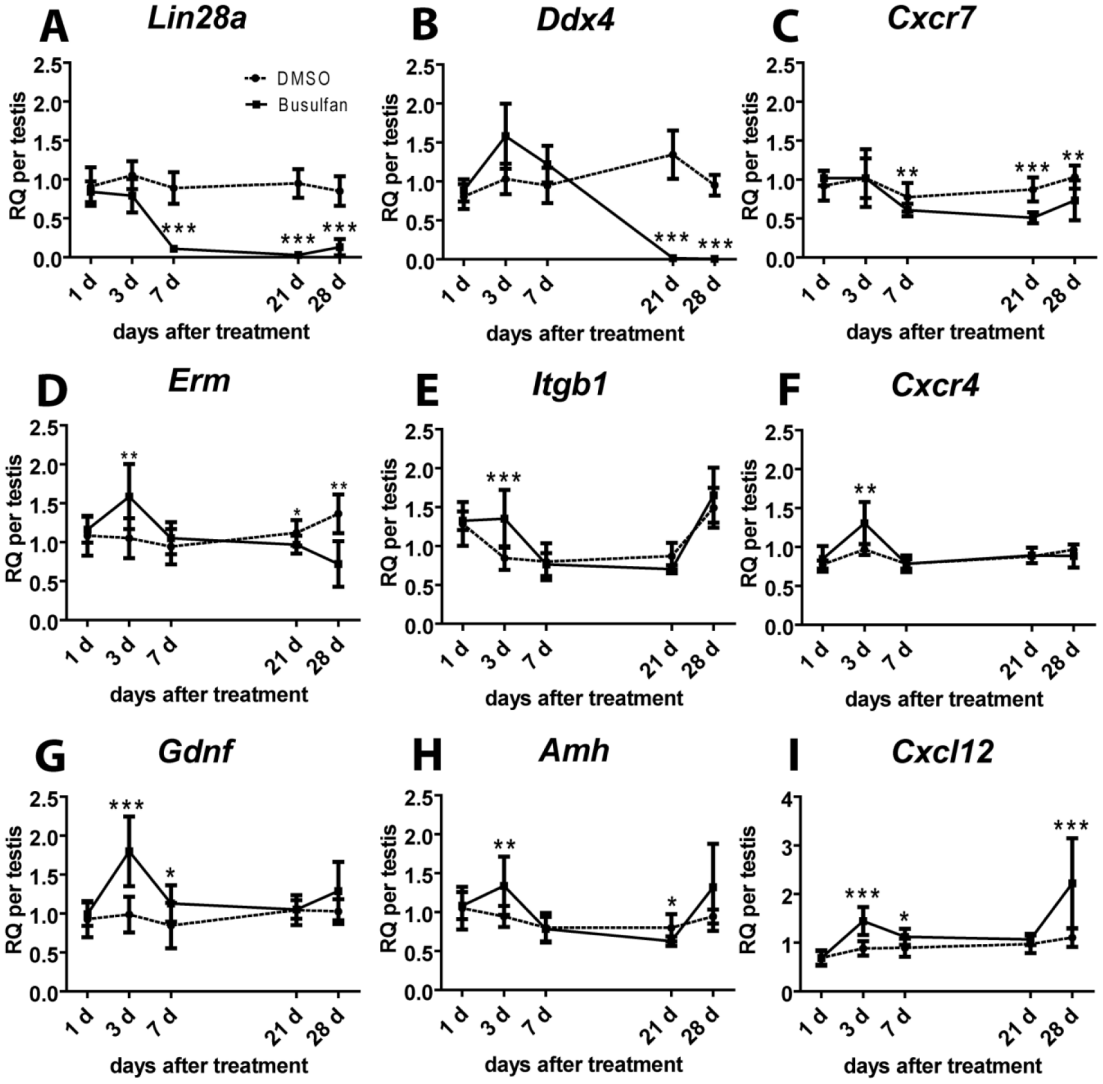
Gene expression patterns of *Lin28a*, *Ddx4* and of SSC niche-associated factors in adult mouse testes after cytotoxic treatments. Changes in transcript levels of *Lin28a* (A), *Ddx4* (B), *Cxcr7* (C), *Erm* (D), *Itgb1* (E), *Cxcr4* (F), *Gdnf* (G), *Amh* (H) and *Cxcl12* (I) on days 1, 3, 7, 21 and 28 after DMSO (•) and busulfan (▪, 38 mg/kg) treatment (n = 10 per time point and treatment). Results were calculated using luciferase as external standard and values from the sham-treated control group (n = 8) as calibrator and are presented as fold change in gene expression per testis (RQ per testis). Significant differences between the DMSO and the busulfan groups are marked with asterisks (*, P = 0.01 to 0.05; **, P = 0.001 to 0.01 or ***, P<0.001).

The progressive germ cell loss is reflected at the transcript level. qRT-PCR analyses showed significantly reduced transcript levels of the spermatogonial marker gene *Lin28a* on days 7, 21 and 28 (Fig. 4A) and the general germ cell marker gene *Ddx4* (Fig. 4B) after 21 and 28 days post busulfan treatment compared to DMSO controls. In line with *Lin28a and* compared to DMSO controls, the transcript levels of *Cxcr7* were also significantly lower on days 7 (1.3-fold), 21 (1.7-fold) and 28 (1.4-fold, Fig. 4C) after busulfan treatment. In contrast to *Cxcr7* and compared to DMSO controls, transcript levels of *Cxcr4* (1.4-fold, Fig. 4F) and of other niche factors (*Itgb1*∶1.6-fold, Fig. 4E; *Gdnf:* 1.8-fold, Fig. 4G; *Amh:* 1.4-fold, Fig. 4H) were significantly higher on day 3. Finally, the mRNA levels of the chemokine *Cxcl12* were significantly higher on days 3 (1.6-fold) and 28 (2.0-fold, Fig. 4I) compared to the DMSO controls.

### Cxcr7 and Cxcr4 expression in seminiferous tubules 28 days after gonadotoxic treatment

**Figure 5:**
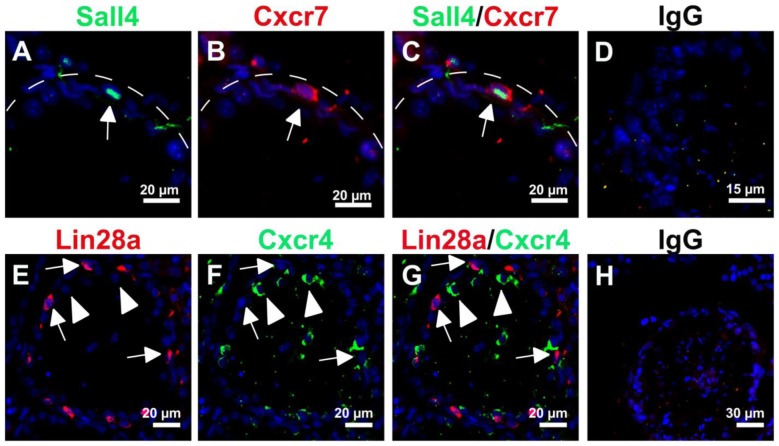
Cxcr7 and Cxcr4 expression in germ cell-depleted mouse testes. Representative immunofluorescence images of testicular tissue section 28 days after busulfan treatment of adult mice. Cxcr7 expression (B, red, arrow) is restricted to Sall4-positive spermatogonia (A, green, arrow) but Cxcr4 expression (F, green, arrowheads) is not solely observed in Lin28-positive spermatogonia (E, red, arrows). The respective merged images are shown in (C) and (G). Hoechst (blue) was used as nuclear counterstain. Incubation with corresponding IgG antibodies was used as negative control and a representative image is shown in (D, H). Scale bars represent 15, 20 and 30 µm.

28 days after busulfan treatment, Cxcr7-positive as well as Cxcr4-positive cells were observed in some seminiferous tubules. Cxcr7 expression was observed in Sall4-positive spermatogonia (Fig. 5A–D). Lin28a-positive spermatogonia repopulating the seminiferous tubules 28 days post busulfan exposure were negative for Cxcr4 as shown by co-localization (Fig. 5E–H).

## Discussion

In the present study, we describe the expression pattern of Cxcr7 during postnatal testicular development and demonstrate that spermatogonial depletion and repopulation has effects on the Cxcl12/Cxcr4/Cxcr7 axis in adult mouse testes.

So far studies in mammalian testes have focused on the tissue distribution and interaction of Cxcl12 and Cxcr4 in embryonic, neonatal and adult mice [14], [15], [19], [20], [37]. In line with our results, Pellegrino *et al.* (2012) have recently demonstrated mRNA expression of *Cxcl12* during various phases of testicular development in mice. In addition, stainings revealed protein expression in the cytoplasm of Sertoli cells in 3 d*pp*, 6 d*pp* and adult mice [13], [15]. Our finding that Cxcr4 transcript expression can be detected throughout testicular development is in general agreement with previous studies. In neonatal mice, gonocytes as well as undifferentiated spermatogonia express Cxcr4, and its expression has also been described in adult mouse testes [13]–[15].

So far, results regarding the localization of the Cxcr4 protein in the adult mouse testis are controversial [14]–[16]. Whereas Yoon *et al.* 2009 localized Cxcr4 in spermatogonia, Sertoli and interstitial cells [16], Kanatsu-Shinohara *et al.* 2012 and Yang *et al.* 2013 localized Cxcr4 only in undifferentiated spermatogonia [14], [16] using co-staining against Plzf or Id4 for characterization of spermatogonia. Our stainings exclusively showed Cxcr4 expression in testicular cells residing along the basement membrane. However, in contrast to Kanatsu-Shinohara *et al.* 2012 and Yang *et al.* 2013, co-expression analysis with the spermatogonial marker Lin28a and Cxcr7 did not provide evidence for co-expression of Cxcr4 in undifferentiated spermatogonia [14], [15], [38], [39]. Therefore, the unequivocal identification of Cxcr4-positive cells in adult NMRI mice remains to be elucidated. Compared to Kanatsu-Shinohara et al. 2012 and Yang et al. 2013, possible reasons for the different results include the use of a different mouse strain and a different fixation agent.

Unexpectedly, we found that the atypical chemokine receptor Cxcr7 is expressed by Sall4-positive spermatogonia throughout testicular development [40]. As this receptor is expressed by somatic cells during PGC migration and acts as a scavenger for Cxcl12 [21]–[24], the germ cell-specific expression was unexpected. However, recent studies suggest, that CXCR7 has diverse cell type-specific functions. Using human vascular smooth muscle cells, it has been demonstrated that CXCR7 can act as an endogenous ß-arrestin-biased signaling receptor to induce cell migration [41]. In addition, CXCR7 may also serve as a co-receptor for CXCR4 and therefore enhance the classical CXCL12-mediated G-protein signaling, including cell migration [42]–[44]. Whether these mechanisms play a role in Cxcr7 expressing germ cells during the postnatal development in mouse testes, however, remains to be elucidated.

In the second part of our study, the effect of busulfan treatment and subsequent germ cell loss on SSC-niche associated factors, including the Cxcl12/Cxcr4/Cxcr7 axis was investigated. Interestingly, previous studies already investigated the impact of busulfan administration of the somatic environment in adult mouse testes and showed that the numbers of Leydig cells and Sertoli cell were not decreased and therefore changes in transcript levels per testis are therefore a reflection of changes per Sertoli cell or Leydig cell, respectively [32]. Regarding germ cells, our histological analyses and immunohistochemical stainings revealed that the seminiferous tubules were sporadically repopulated with Lin28a-positive spermatogonia 28 days after busulfan treatment, supporting previous findings demonstrating spermatogonial repopulation to be initiated as early as day 15 after busulfan administration (dose-dependent) [45], [46]. Furthermore, we demonstrated that Ddx4-positive spermatocytes and spermatids were absent from the tubules between days 7 and 21 after treatment. Performing qPCR analyses for *Lin28a* and *Ddx4*, we demonstrated that the loss of the respective testicular cell type is also reflected at the RNA level. In line with our results, O'Shaughnessy et al. (2008) showed that transcript levels of *Stra8* (spermatogonia associated gene) and of *Spo11* (spermatocytes associated gene) decreased on day 5 and day 15 after busulfan administration, respectively [32]. These findings revealed that RNA expression analysis for specific marker genes is a valid tool to evaluate changes regarding the composition of testicular cell types. Interestingly, despite the fact that busulfan treatment had not yet resulted in a loss of all spermatogonia on day 3 after treatment, we found that expression levels of the ligand *Cxcl12* and its receptor *Cxcr4* as well as of the growth factors *Amh*, *Gdnf* and the transcription factor Erm were significantly increased compared to the vehicle control (DMSO). Consistent with our findings, previous studies have shown a similar increase of *Gdnf* transcripts during the loss of spermatogonia following gonadotoxic treatments [47], [48]. Zhoni et al., (2011) and Ventelä et al. (2012) reported a 2-fold increase of *Gdnf* transcript levels 5 days after busulfan treatment and 24 hours after X-irradiation, respectively. Both studies suggested that Sertoli cells respond rapidly and temporarily to the loss of spermatogonia and support the survival of remaining germ cells and stimulate their proliferation [48], [49]. Moreover, our data reveal significantly increased expression levels of the chemokine Cxcl12 at the time of the early spermatogenic repopulation (on day 28 after busulfan treatment). These results are in line with previous reports, showing that most changes in transcript levels of 26 Sertoli cell-specifc genes were associated with the loss of the last spermatids and the early time of spermatogenic repopulation [32]. The expression pattern of Cxcr4, which was not detected on Lin28a-positive or Cxcr7-positive spermatogonia, was reflected at the RNA level following busulfan treatment, as transcript levels of Cxcr4 remained rather constant. In contrast to Cxcr4, we found that transcript levels of the chemokine receptor Cxcr7 were decreased following day 3 after busulfan treatment. The expression pattern therefore rather resembles that of the spermatogonial marker gene Lin28a and is consistent with our protein data demonstrating that Cxcr7 is expressed by germ cells located at the basement membrane in normal adult mouse testes.

However, all studies revealing an intense loss of post-meiotic germ cells suffer from the fact that relative changes in somatic cell mRNA levels may be overestimated. In such scenarios any detectable change could simply reflect the fact that Sertoli cell transcription is unaffected while germ cell mRNA is diminished due to loss or inactivity of germ cell transcription.

Interestingly, performing immunofluorescence analyses on day 28 after busulfan treatment, we again observed Cxcr4 expression only in Lin28a-negative testicular cells. Furthermore, analysis on day 28 after busulfan treatment showed the expression of Cxcr7 on spermatogonia which repopulate seminiferous tubules. These findings were unexpected as the expression of Cxcr7 was only detected in somatic cells during germ cell migration of zebrafish [21], [22]. Our findings therefore suggest an additional function of Cxcr7 in mouse germ cells at a) later developmental stages and b) during testicular recovery in the mouse testis. Investigating the functional role of CXCR7 in human cell lines (HEK-293T and MDA-MB-231) two mechanisms have been suggested regarding the CXCL12 mediated action of CXCR7. These include active signalling [50], [51] and the modulation of CXCR4 activity via heterodimerization [42], [43], [52]. Interestingly, it was also demonstrated that cells showed enhanced growth and survival as well as adhesion properties following ligand binding to CXCR7 [53]. However, whether these mechanisms are relevant to the germ cell population in mammalian testes remains to be elucidated.

So far, studies in mammalian testes have focused on the interaction of Cxcl12 and Cxcr4 [14], [15]. We demonstrate for the first time that the chemokine receptor Cxcr7 is expressed throughout postnatal testicular development and recent studies suggest that this receptor may not act solely as a scavenger receptor for Cxcl12 but may rather be involved in the regulation of SSC growth and survival as well as cell adhesion [53]. Moreover, we suggest that the interaction between Cxcl12 and Cxcr7 may stimulate the spermatogenic repopulation of the seminiferous tubules following depletion. Further support for this suggestion is provided by the observed expression of the chemokine Cxcl12 during re-colonization of murine SSCs following germ cell transplantation [14]. Furthermore, we demonstrated that those germ cells repopulating the seminiferous tubules after induced germ cell depletion are immunopositive for Cxcr7. Our study therefore re-inforces the complexity of this system and future studies will aim to elucidate the functional role of the Cxcl12/Cxcr4/Cxcr7 interaction during testicular development and recovery.

## Supporting Information

Figure S1 **Testis weights following busulfan treatment.** Adult mice were given a single injection of DMSO (•) or busulfan (▪ 38 mg/kg) and testis weights were measured on days 1, 3, 7, 21 and 28 (n = 10 per time point) after treatment. Results are expressed as mean ± SD. Busulfan groups marked with asterisks are significantly different (*, P = 0.01 to 0.05; **, P = 0.001 to 0.01 or ***, P<0.001).
(TIF)

Figure S2 **Cxcr7 is expressed by undifferentiated spermatogonia during testicular germ cell development.** The specific staining of the anti-CXCR7 antibody was validated by a peptide blocking experiment (A–C). Cxcr7 expression is restricted to Sall4-positive undifferentiated spermatogonia on days 7 (E–G) and 14 (I–K) of postnatal testicular development and in adult tissue (M–O). Incubation with corresponding IgG antibodies was used as negative control and a representative image is shown in (D, H, L, P). Scale bars represent 20 µm and 30 µm.
(TIF)

Figure S3 **Representative micrographs of adult mouse testes after cytotoxic treatments.** Micrographs showing PAS stained sections of testicular tissues on days 1 (A), 3 (B), 7 (C), 21 (D) and 28 (E) following DMSO injections. In contrast to the DMSO treatment group, which showed no depletion of germ cells, the testicular parenchyma of the busulfan treatment group still contained all germ cell types and Sertoli cells (arrowheads) on days 1 and 3 (F, G; Insert: K, L) but had lost spermatogonia (arrows) on day 7 (H; Insert: M)). Furthermore, spermatocytes were lost after 21 days (I; Insert: N) and on day 28 most tubules showed a Sertoli cell-only phenotype, with individual tubules containing spermatogonia (arrows) (J; Insert: O). Scale bars represent 10 µm and 50 µm.
(TIF)

Table S1 **Primer sequences used for real-time PCR, localization of respective proteins in the mouse testis and corresponding references.**
(DOCX)

Table S2 **Qualitative assessment of the Cxcr7 expression in mouse testes during testicular germ cell development.**
(DOCX)
