# Supplementary figures and images for: Profiling of Cxcl12 Receptors, Cxcr4 and Cxcr7 in Murine Testis Development and a Spermatogenic Depletion Model Indicates a Role for Cxcr7 in Controlling Cxcl12 Activity
Source: PLoS One. 2014 Dec 2;9(12):e112598. doi: 10.1371/journal.pone.0112598 (PMC4251904; doi:10.1371/journal.pone.0112598)

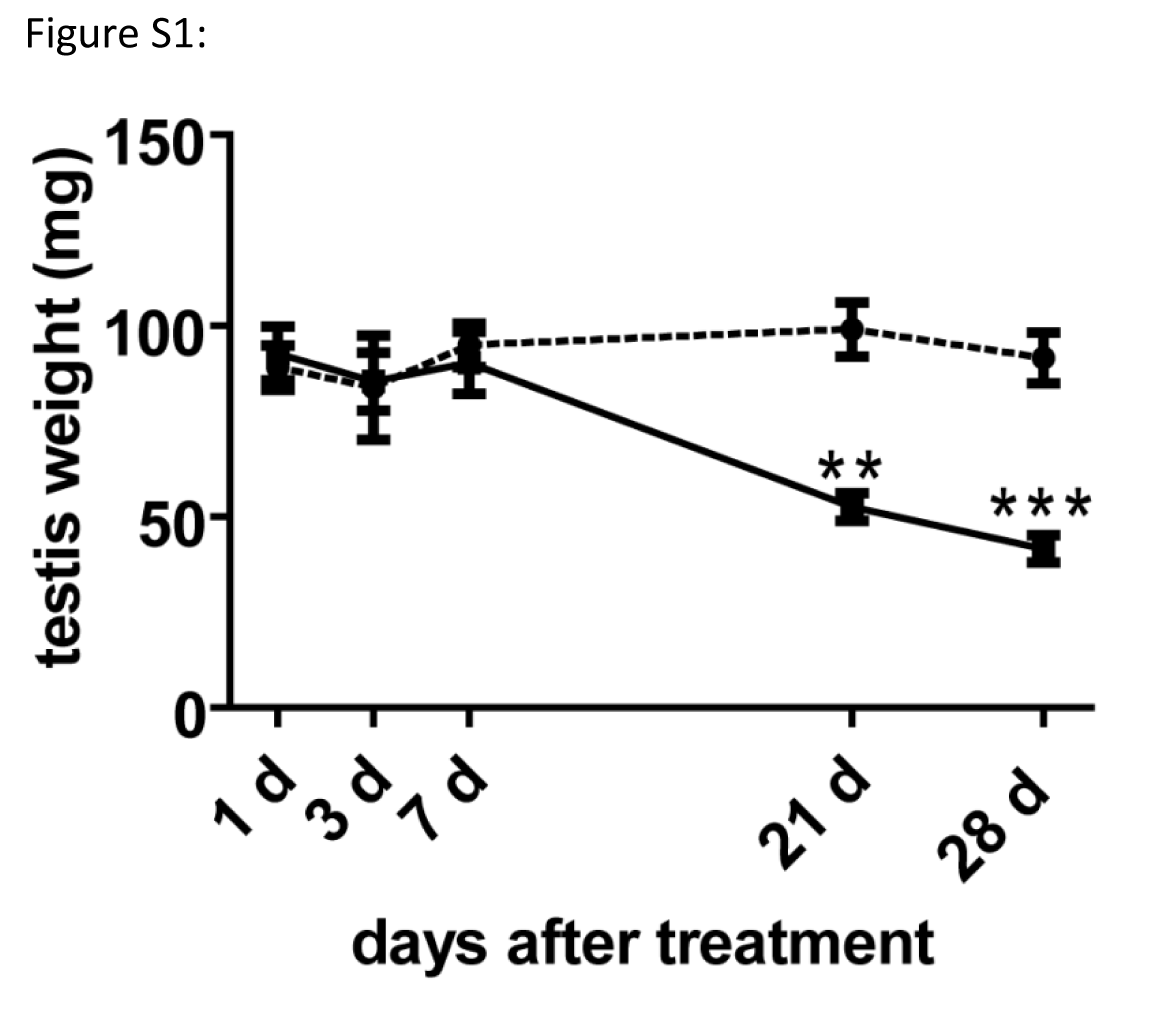

Supplement: Figure S1 — Testis weights following busulfan treatment. Adult mice were given a single injection of DMSO (•) or busulfan (▪ 38 mg/kg) and testis weights were measured on days 1, 3, 7, 21 and 28 (n = 10 per time point) after treatment. Results are expressed as mean ± SD. Busulfan groups marked with asterisks are significantly different (*, P = 0.01 to 0.05; **, P = 0.001 to 0.01 or ***, P<0.001). (TIF) [file pone.0112598.s001.tif]

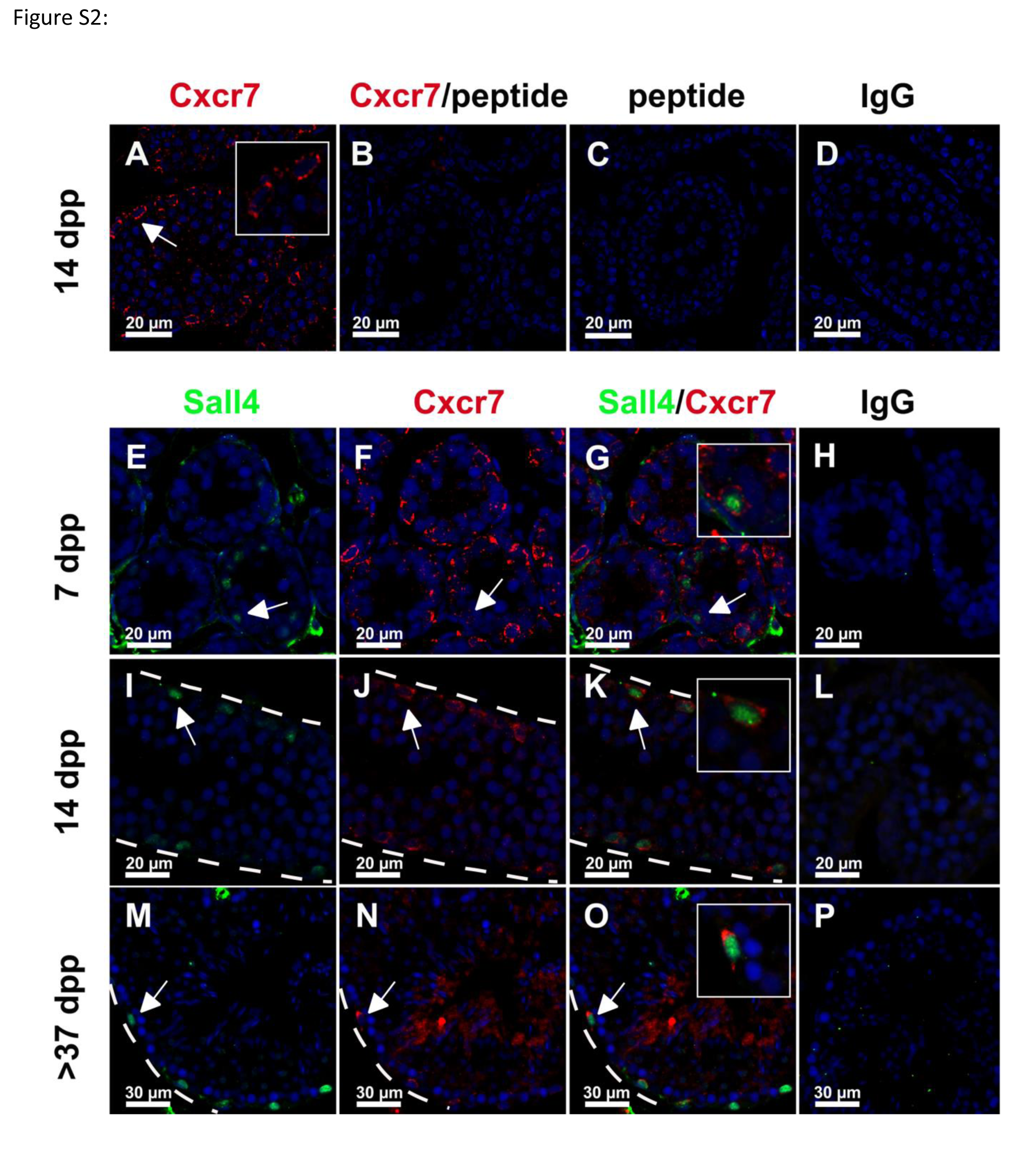

Supplement: Figure S2 — Cxcr7 is expressed by undifferentiated spermatogonia during testicular germ cell development. The specific staining of the anti-CXCR7 antibody was validated by a peptide blocking experiment (A–C). Cxcr7 expression is restricted to Sall4-positive undifferentiated spermatogonia on days 7 (E–G) and 14 (I–K) of postnatal testicular development and in adult tissue (M–O). Incubation with corresponding IgG antibodies was used as negative control and a representative image is shown in (D, H, L, P). Scale bars represent 20 µm and 30 µm. (TIF) [file pone.0112598.s002.tif]

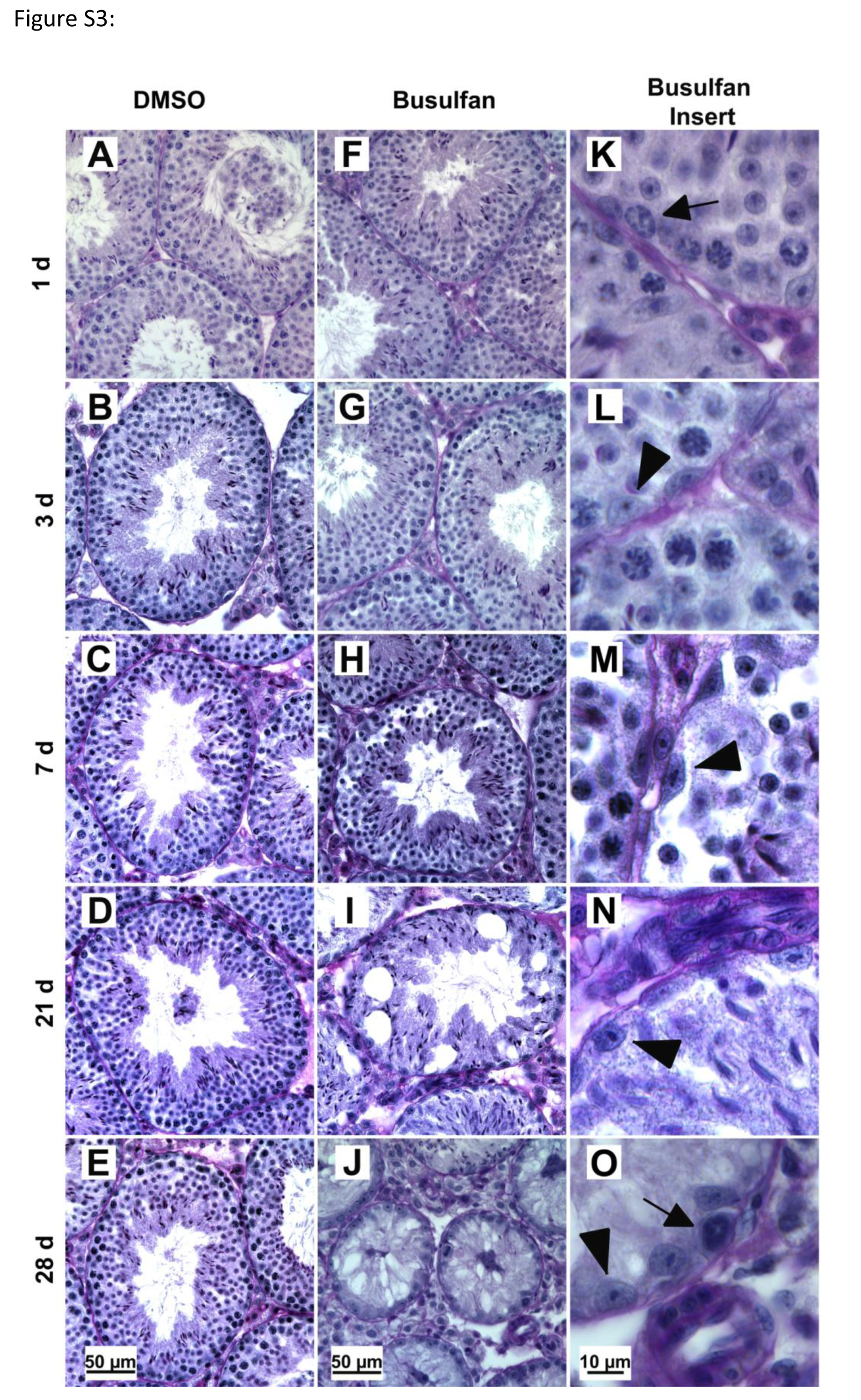

Supplement: Figure S3 — Representative micrographs of adult mouse testes after cytotoxic treatments. Micrographs showing PAS stained sections of testicular tissues on days 1 (A), 3 (B), 7 (C), 21 (D) and 28 (E) following DMSO injections. In contrast to the DMSO treatment group, which showed no depletion of germ cells, the testicular parenchyma of the busulfan treatment group still contained all germ cell types and Sertoli cells (arrowheads) on days 1 and 3 (F, G; Insert: K, L) but had lost spermatogonia (arrows) on day 7 (H; Insert: M)). Furthermore, spermatocytes were lost after 21 days (I; Insert: N) and on day 28 most tubules showed a Sertoli cell-only phenotype, with individual tubules containing spermatogonia (arrows) (J; Insert: O). Scale bars represent 10 µm and 50 µm. (TIF) [file pone.0112598.s003.tif]
