# Supplementary material for: Profiling of Cxcl12 Receptors, Cxcr4 and Cxcr7 in Murine Testis Development and a Spermatogenic Depletion Model Indicates a Role for Cxcr7 in Controlling Cxcl12 Activity
Source: PLoS One. 2014 Dec 2;9(12):e112598. doi: 10.1371/journal.pone.0112598 (PMC4251904; doi:10.1371/journal.pone.0112598)
Supplement: Table S1 — Primer sequences used for real-time PCR, localization of respective proteins in the mouse testis and corresponding references. (DOCX) [file pone.0112598.s004.docx]

**Table S1:**

| **Target** | **Accession number** | **Forward Primer (5´- 3´)** | **Reverse Primer (5´- 3´)** | **Localization in the testis (mouse)** | **References** |
| --- | --- | --- | --- | --- | --- |
| ***Amh*** | NM_007445 | GGGCCTCATCTTAACCCTTCA | AAGGCTTGCAGCTGATCGAT | Sertoli cells (prepubertal) | [47] |
| ***Cxcl12α*** | NM_021704 | CAGTGACGGTAAACCAGTCAGC | TGGCGATGTGGCTCTCG | Sertoli cells | [13,14,48] |
| ***Cxcr4*** | NM_00911 | TCCTCCTGACTATACCTGACTTCATCT | CCTGTCATCCCCCTGACTGAT | Spermatogonia, Sertoli cells | [16] |
| ***Cxcr7*** | NM_001271609 | AACCTCTTTGGGAGCATCTTCTT | GGTGCCGGTGAAGTAGGTGAT | unknown | unknown |
| ***Ddx4*** | NM_001145885 | CATCTGTTGACACGAGGAAGAATT | GAAGAAGAAATCCCCGCTGTATT | Spermatocytes, round spermatids | [49] |
| ***Erm*** | NM_023794 | CTCCAGCAGCCATGAAGGA | GGTACCACGCAAGTAATCATCAAAG | Sertoli cells | [50,51] |
| ***Gdnf*** | NM_010275 | TGTCTCGGAGTAGAAGGCTAACAA | CGACCGGCCTGCAACA | Sertoli cells | [8,52] |
| ***Lin28a*** | NM_145833 | GGTGGTGTGTTCTGTATTGGGA | AGTTGTAGCACCTGTCTCCTTTG | Undifferentiated spermatogonia | [29] |
| ***Luciferase**** | # | GCACATATCGAGGTGAACATCAC | GCCAACCGAACGGACATTT | # | [26] |

*** Primers for luciferase were designed based on the mRNA sequence provided by Promega**
