# Supplementary material for: Profiling of Cxcl12 Receptors, Cxcr4 and Cxcr7 in Murine Testis Development and a Spermatogenic Depletion Model Indicates a Role for Cxcr7 in Controlling Cxcl12 Activity
Source: PLoS One. 2014 Dec 2;9(12):e112598. doi: 10.1371/journal.pone.0112598 (PMC4251904; doi:10.1371/journal.pone.0112598)
Supplement: Table S2 — Qualitative assessment of the Cxcr7 expression in mouse testes during testicular germ cell development. (DOCX) [file pone.0112598.s005.docx]

**Table S2:**

| **d*pp*** | **IC** | **SC** | **GC** | **Spg** | **Spc** | **RS** | **ES** |
| --- | --- | --- | --- | --- | --- | --- | --- |
| **1** | + | - | + | - | - | - | - |
| **7** | + | - | - | + | - | - | - |
| **14** | + | - | - | + | + | - | - |
| **21** | + | - | - | + | + | - | - |
| **> 37** | + | - | - | + | - | - | + |

**Abbreviations stand for:** d*pp* = days *post partum*; IC = interstitial cells; SC = Sertoli cells; GC = Gonocytes; Spg = Spermatogonia; Spc = Spermatocytes; RS = Round spermatids; ES = Elongated spermatids.
